# Community out-of-hours palliative care – ‘It’s a patchwork of services’: A qualitative study exploring care provision

**DOI:** 10.1177/02692163241302671

**Published:** 2024-12-11

**Authors:** Alice M Firth, Joanna Goodrich, Inez Gaczkowska, Richard Harding, Fliss EM Murtagh, Catherine J Evans

**Affiliations:** 1 Department of Palliative Care, Policy and Rehabilitation, Cicely Saunders Institute of Palliative Care Policy & Rehabilitation, London, UK; 2 Wolfson Palliative Care Research Centre, University of Hull, Hull, UK

**Keywords:** After-hours care, palliative care, community health services, models organisational

## Abstract

**Background:**

People in receipt of community palliative care usually receive care from a range of services and require access to care 24/7. However, care outside of normal working hours varies, with little understanding of which models of care are optimal.

**Aim:**

To identify and characterise current models of out-of-hours community palliative care in the UK and explore healthcare professionals’ views on the barriers and facilitators to providing high quality community out-of-hours care.

**Design:**

Exploratory qualitative study using semi-structured interviews, analysed using reflexive thematic analysis.

**Setting and participants:**

We recruited 39 healthcare professionals from 20 geographic areas. Participants were service leads from community palliative care, district/community nursing and primary care providers.

**Results:**

Four overarching models of out-of-hours palliative care identified, characterised by levels of integration between services, balance between generalist and specialist providers, availability of care and type of care provided (hands-on clinical care/ advisory care). Analysis of barriers and facilitators generated three themes: (1) ‘It’s never one service’: challenges of coordination of care across multiple services, (2) Need for timely skilled management of distressing symptoms, (3) ‘We’re just plugging gaps’: prioritising patient care within limited resources. Patterns within the themes varied across the four models.

**Conclusion:**

This study identifies key characteristics of four common models of out-of-hours palliative care, from the perspectives of professionals. Facilitators of high quality out-of-hours care include: a palliative care specific single point of access for patients; formal structures to integrate generalist/specialist services; and timely/skilled management of symptoms. We provide recommendations for a potential model incorporating these factors.

**What is already known about the topic?**

- Improving the provision of palliative care ‘out-of-hours’ is a priority for patients and families.
- There is global variation in the provision of out-of-hours palliative care.
- There is evidence of improvement in some patient outcomes with the provision of 24/7 specialist palliative care that provides both advisory and hands-on nursing care.

**What this study adds?**

- We identify four models of out-of-hours community palliative care characterised using: balance of provision between generalist and specialist community services, the type of care (advisory and hands-on nursing care) provided, the hours of availability of the care for example, 24/7, 6 pm–7 am or 5 pm–midnight, etc. and the level of integration between services.
- We identify three themes to understand the barriers and facilitators of out-of-hours care provision: (1) ‘It’s never one service’: challenges of coordination of care across multiple services, (2) Need for timely skilled management of distressing symptoms, (3) ‘We’re just plugging gaps’: prioritising patient care within limited resources.
- We identify a model of care which facilitates high quality community out-of-hours care from healthcare professionals’ perspectives. The model provides patients and families with a single point of access, staffed by an experienced palliative care clinician, who coordinates the individual’s care across all involved services.

**How this study might affect research, practice or policy**

- This paper proposes a model of palliative care which professionals describe as facilitating high quality out-of-hours care.
- When designing out-of-hours care models, policy makers and healthcare service providers should prioritise formal and informal structures that foster efficient integration and coordination of care within and between existing generalist and specialist services.
- Further research is needed to understand how the identified models of care impact the patients and families experience.

## Background

People and families receiving community-based palliative care frequently rely on ‘out-of-hours’ services.^1–3^ Out-of-hours healthcare constitutes 63% of the week and is defined as healthcare provided outside of core working hours, (i.e. evenings, nights, weekends and public holidays).^4^ This provision is highly variable in terms of: hours available, mode of care delivery (telephone or in-person), staff providing care and types of intervention.^5–8^ However, people with advanced disease experience a range of distressing symptoms.^9^ and for these patients to be able to remain at home they require palliative care which is available 24 h a day and 7 days a week (24/7).^10–16^

Striving to provide high quality care is intrinsic to the nature of palliative care.^17^ High quality care is a multi-dimensional and complex. The World Health Organization defines quality of care as ‘the degree to which health services for individuals and populations increase the likelihood of desired health outcomes’.^18^ Additionally, high quality care should be effective, acceptable and efficient.^19^ A recent consensus study showed that patients and professionals view having an accessible and responsive district nursing service and the availability of medicines to relieve suffering as essential components to high quality out-of-hours palliative care.^20^

Internationally, out-of-hours care is provided by different services such as primary care, physicians, community nursing and specialist palliative care teams. Community specialist palliative care services offer patients with life-limiting illness and their families, assessments, ongoing care and personal care. These teams work in partnership with community nurses, pharmacists, medical teams, general practitioners and social services to provide out-of-hour services. Our previous international systematic review developed a typology for individual services to define when, what and how out-of-hours community palliative care is provided.^14^ However, there is limited research which identifies the roles, relationships and interactions of the different services which contribute to providing out-of-hours care or what environmental factors facilitate or prevent the delivery of high-quality care. Therefore, in this paper, we aimed to identify and characterise current models of out-of-hours community palliative care in the UK and to explore healthcare professionals’ views on the barriers and facilitators to providing high quality community out-of-hours care to patients in receipt of palliative care.

## Methods

This paper forms part of a wider study on out-of-hours community palliative care, which includes a systematic review forming a typology of out-of-hours care^14^ and a Delphi study to identify the priority components of out-of-hours care.^20^

### Design

Exploratory qualitative interview study underpinned by Reflexive Thematic Analysis.^21^ This approach allowed for in-depth exploration of the different models of care across the UK and understanding of facilitators and barriers to out-of-hours care from the perspectives of healthcare professionals. Reflexive Thematic Analysis reporting guidelines (RTARG)^22^ were followed for the reporting of this study.

### Theoretical underpinning

This study uses Pask et al.’s^23^ adaption of Bronfenbrenner’s Ecological Systems Theory to understand complexity in palliative care. Pask’s et al.^23^ modification of this framework explains how a patient interacts with their context and environment when living with an advanced illness. We use this adapted framework to explore the dynamic interactions of community out-of-hours palliative care.

### Setting

Nine community specialist palliative care teams were existing research sites for the wider project and a further 11 community specialist palliative care teams were recruited by public advert. All sites were purposively sampled by geographic location (across the 10 Palliative and End of Life Care Strategic Clinical Networks, and Wales, Scotland and Northern Ireland) once.

### Participant eligibility

Eligible participants comprised of healthcare professionals from or working with the research site who were involved in the provision of out-of-hours community palliative care. Thisncluded NHS community palliative care or charitable hospice services, primary care/family practice and community and district nursing teams with representation from across the multi-disciplinary team.

### Recruitment

Research leads from the respective study sites identified and approached a range of healthcare professionals in their geographic area involved in leading out-of-hours community services for palliative care patients to participate in the study and provided potential participants with an information sheet. This included, specialist palliative care staff, district nurses, GP’s and managers and service leads. The researchers (AF and JG) then approached those expressing interest, for consent and interview.

### Sampling

Our achieved sample size of 39 was guided by the concept of Information power which relates the adequacy of a sample size to the specificity of a study aim, the sample diversity and the quality of data collected.^24^ This study had a specific aim of exploring experiences of providing out-of-hours care, with professionals all working in community palliative care services through in-depth interviews with experienced researchers. Sampling closed when sufficient representation from each geographical location and the different types of healthcare professional involved in out-of-hours care.

### Data generation

Semi-structured qualitative individual or small group interviews (two or three staff from the same geographic location) conducted online using MS-Teams or Zoom. Interviews were undertaken between September 2021 and January 2022. The interview topic guide (Supplemental Material 1) was informed by the preceding studies and underpinning theory and co-designed with the project patient and public involvement group.^14,20,23^ In the interview, participants were asked to describe how out-of-hours care was provided in their locality for patients and families in receipt of specialist palliative care. Then, to describe the barriers and facilitators encountered to provide high quality out-of-hours community care. AF and JG, who are experienced qualitative researchers with backgrounds in sociology and palliative care, conducted the interviews. The interviews ranged from 20 to 70 min (mean = 48 min). They were digitally recorded with permission, transcribed verbatim, checked for accuracy and anonymised.

### Ethics committee

London Bloomsbury Research Ethics Committee reference: 19/LO/1865.

### Data analysis

Data analysis was conducted in two stages: (1) identifying and characterising models of out-of-hours care; and (2) Using reflexive thematic analysis to explore the barriers and facilitators of out-of-hours care.

#### Stage 1: Identifying and characterising current models of out-of-hours care

Deductive data analysis using a matrix constructed from the preceding systematic review.^14^ The review detailed a typology which provided dimensions and components to define individual out-of-hours service available.^14^ Dimensions included: (1) service times, (2) the focus of the team delivering the care and (3) the type of care delivered (advisory and/or hands-on clinical care). Data were extracted systematically from each interview transcript about the respective services described. Additional components or descriptors of the services were added inductively to the matrix.

The researchers then used the matrix detailing the different services available to patients and families in one geographic area to identify similarities and differences, and to identify and distinguish models of care. An expert panel was used to review the dimensions selected to form the models of care and then to sense check the identified models of care and provide feedback The expert panel included family carers, palliative care practitioners, healthcare commissioners and researchers in palliative care and experienced by practice or experience in different model of out-of-hours care.

#### Stage 2: Reflexive thematic analysis to explore the barriers and facilitators of providing different models of out-of-hours care

Inductive reflexive thematic analysis was used to develop, analyse and interpret patterns within the data.^25^ Interviews were analysed (by AF, JG and IG) in NVivo V1.6.1. Reflective thematic analysis allows for an inductive approach and the identification of new themes to be developed from the new data generated with healthcare professionals. Reflexive thematic analysis also allowed for the comparisons of how themes applied between models of care. Pask et al.’s 2018 work underpinned our conceptual understanding with the patient and family considered to be at the centre of the model of care they were receiving, surrounded with many levels of complexity, including how systems and services interact to provide care.^23^

Data analysis followed the six phases of reflexive thematic analysis.^21^ Firstly two researchers (AF and JG) familiarised themselves with the data through revisiting the audio recordings, and transcripts, The initial codes were generated to capture meaningful basic elements of the data in relation to the study objectives by AF and JG. Codes were then discussed and defined by AF and JG. Meanings were primarily considered at a semantic (explicit) level, but with consideration of latent (implicit) interpretations. Themes were generated by discussing, reviewing, refining and grouping codes and writing definitions accompanied by illustrative quotes until consensus between AF and JG was reached. Themes were discussed and revised with the wider project team (FM, CE and RH) and patient and public involvement group, this provided representation from academic, clinical and service user experiences. Using NVivo framework matrices, we explored convergence and divergence of the generated themes across models of care identified in the stage 1 analysis. The themes were discussed, developed and debated until the research team were happy that interpretations of data accurately reflected the different models and participant accounts.

In terms of positionality, team members AF, JG, RH, FM and CE had all been involved in the wider study on out-of-hours care^14,20,26^ and the outputs of these elements influenced the construction and generation of the codes. However, AF and JG also focussed on coding the interview data on barriers and enablers to care inductively to allow the generation of new ideas and concepts.

## Results

Twenty-eight interviews (individual and small group) with 39 participants were completed from 20 research sites across the UK. See Table 1 for professional and geographic details. The interviews ranged from 20 to 70 min (mean = 48 min).

**Table 1.** Role and region of interview participants.

Health care participants specialty and primary role	*N* = 39
Specialist palliative care lead – Doctor	6
Specialist palliative care lead – Nurse	15
Dedicated palliative care service lead – Nurse	6
District or Community Nurse (generalist)	6
Out-of-hours Family practice/ General Practitioner (generalist)	3
Managers and commissioners (specialist PC and/or generalists)	3
Geographic location of healthcare participants workplace using the UK Palliative and End of Life Care strategic Clinical Networks and UK devolved nations *N* = 39	
England - North East and Yorkshire	10
England - North West	2
England – Midlands	2
England - East of England	4
England – London	2
England - South East	7
England - South West	4
Wales	3
Northern Ireland	1
Scotland	4

### Stage 1 Results: Identifying and characterising different models of out-of-hours care

We identified many services involved in providing out-of-hours care, but there was significant variation in terms of which services recipients across the UK had access to. Combined, these services constituted an identifiable ‘model of care’ for patients and families that could include specialist palliative care teams (NHS or charitable); charitable nursing teams; district or community nursing teams; and support from primary care.

**Figure 1.**
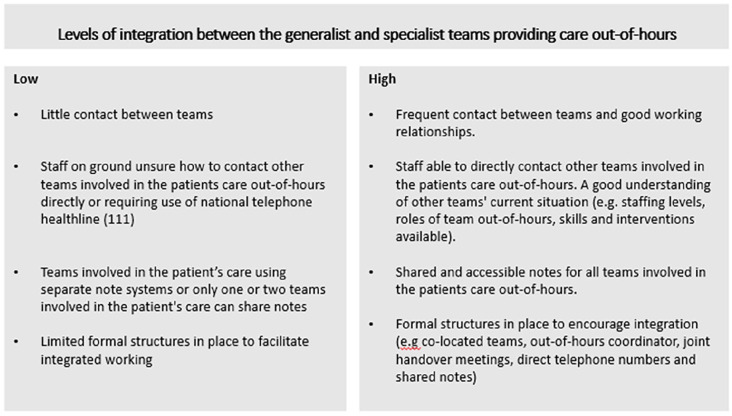
Defining level of integration between teams providing out-of-hours community palliative care.

**Figure 2.**
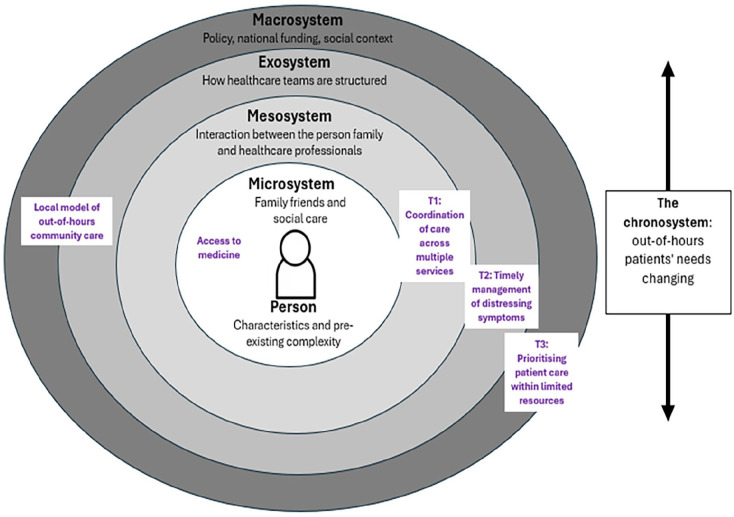
Application of Pask et al.’s^23^ adaption of Bronfenbrenner’s ecological systems theory.

We identified four overarching models of out-of-hours care, characterised by (1) high or low levels of integration between services (see Figure 1), (2) the balance between generalist and specialist care and (3) the type of care available (hands-on or advisory care). We term these models as ‘overarching’ because of the variation in the services provided within each model (see Table 2 for overview, and Table 3 for details).

- Model A is heavily reliant on GP out-of-hours care. There is some specialist palliative care/dedicated palliative care available, but this is mainly advisory. District/community nursing is only available for some of the out-of-hours period.
- Model B was the common model, with numerous services available out-of-hours, including GP and district/community nursing team, advisory specialist/ dedicated palliative care available and some (not 24/7) specialist/dedicated palliative care hands-on nursing care. There are low levels of integration between the services
- Model C provides 24/7 specialist/ dedicated palliative care offering both advisory and hands-on nursing care to patients and families. GP and district/community nursing care is also available. There are low levels of integration between the services.
- Model D is a highly integrated multidisciplinary service via a dedicated 24/7 telephone line staffed by experienced palliative care nurses, supported by community nursing, GP and specialist palliative care services, providing hands-on clinical and advisory care.

**Table 2.** Overview of four models of out-of-hours community palliative care.

• Model A is heavily reliant on GP out-of-hours care. There is some specialist palliative care/dedicated palliative care available, but this is mainly advisory. District/community nursing is only available for some of the out-of-hours period.
• Model B was the common model, with numerous services available out-of-hours, including GP and district/community nursing team, advisory specialist/ dedicated palliative care available and some (not 24/7) specialist/dedicated palliative care hands-on nursing care. There are low levels of integration between the services
• Model C provides 24/7 specialist/ dedicated palliative care offering both advisory and hands-on nursing care to patients and families. GP and district/community nursing care is also available. There are low levels of integration between the services.
• Model D is a highly integrated multidisciplinary service via a dedicated 24/7 telephone line staffed by experienced palliative care nurses, supported by community nursing, GP and specialist palliative care services, providing hands-on clinical and advisory care.

**Table 3.** Characteristics of the Identified Models of Out-of-Hours Palliative Care.

Model identifier	Description of model	Balance of provision between generalist and specialist community services, the type of care (advisory and hands-on nursing care) and the times/availability of the care			Level of Integration (high or low) between generalist and specialist teams providing care out-of-hours (see Figure 1 for definitions)
		When is advisory care available from specialist palliative care?	What hands on clinical care available from specialist or dedicated palliative care?	Family practice/General practitioner and community nursing	
Model A	Specialist/dedicated palliative care but advisory care only out-of-hours, heavily reliant on generalist services out-of-hours	Advisory care available but may not be 24/7	None	Community nursing not 24/7. General practitioner available 24/7	Low
Model B	Some specialist/dedicated palliative care hands on visits at weekends and/or evenings (not 24/7) and some advisory care	Advisory care available but may not be 24/7	Hands on care available for some of the out-of-hours period but not 24/7	Available 24/7	Low
Model C	24/7 advisory and hands on specialist/dedicated palliative care	Advisory care 24/7	Hands on care 24/7	Available 24/7	Low
Model D	Integrated care. A combination of generalist and specialist palliative care services working closely together to provide out-of-hours care.	Generalist and specialist services integrated to provide advisory care	Generalist and specialist services integrated to provide hands on care	Available 24/7	High

### *Stage 2*: Barriers and facilitators to high quality out-of-hours care

These were constructed as three themes (1) ‘It’s never one service’; challenges of coordination of care across multiple services, (2) Need for timely skilled management of distressing symptoms and (3) ‘We’re just plugging gaps’; prioritising patient care within limited resources. Supplemental Material 2 provides further details of the three themes and subthemes.

#### Theme 1: ‘It’s never only one service’; Challenges of coordination of care across multiple services

Participants described the challenges of working with multiple services out-of-hours, which intensified when there was low integration between providers (as in models A, B and C). Services working in the same geographical area often used different electronic patient record systems and therefore did not have access to patient records from other services. For example, the district nurses could view the GP record, but not the specialist palliative care team record, or vice versa. In models A and B, a lack of direct access to GP out-of-hours and community nursing, meant that services were often required to telephone 111 (the UK national non-urgent telephone help line) to obtain care, which often involved long waits for a response. This took up large amounts of staff time, delayed patient care, caused frustrations and prevented good working relationships with other services. In this quote, a district nurse describes trying to contact the GP out-of-hours.

*but often it’s a case of we ring 111 and they say someone will ring back, you wait hours, meanwhile the family are ringing* Model B. Participant 0013: District nurse.

Model C provided a ‘one service approach’ out-of-hours with the palliative care team providing most of the hands-on clinical care and advisory care 24/7. This reduced some of the need for coordinated of care between external services. However, patients, families and the palliative care team still sometimes required other services, such as phoning 111 to access the GP, if the nurse prescribers were unavailable or for certain assessments. Although the need to contact other services was less frequent than other models, it highlighted the importance of integration for all models of care.

*So we have a team of . . .two nurses and two health care assistants [who] will go out and cover emergency visits. But they can also give advice on the phone, which is good.* Model C. Participant 0040: Hospice lead.

One of the defining components of model D was the high level of integration between all the providers involved in a patient’s care out-of-hours. Model D had a single point of contact by telephone which was answered by an experienced palliative care nurse who then coordinated care with other services on behalf of the patient and family. Shared electronic patient records could be accessed and were used by all the providers in that locality. Formal strategies for integration also included providing direct telephone numbers for healthcare professionals to contact other services, having regular meetings between services and clear agreements about the roles of each team.

*the community nursing team, the General District Nursing team, the GP. . . . . . our single point of access and us, we’re all on SystmOne [the same electronic patient record system]. So, we all share that electronic record, and that’s all we use. . .and then we have a shared agreement when the patient comes onto the caseload that what we write will be shared* Model D. Participant 0018: Hospice lead.

Healthcare professionals felt that continuity of care was provided when the patient and family felt known to the service, professionals were able to use shared records between services, directly contact services, care was well planned for and anticipated and when patients and families had one point of access out-of-hours (as described in model D).

#### Theme 2: Need for timely skilled management of distressing symptoms

Community out-of-hours care attempts to ensure that patients and families receive responsive and skilled care for unstable and distressing symptoms. Indeed, the timely management of symptoms was viewed as vital by all healthcare professionals. However, staff reported problems with responding to patient needs due to a lack of dedicated time, which was exacerbated by the number of staff vacancies in out-of-hours services (across all models).

Barriers to achieving a responsive service in model A included palliative care staff not having dedicated or sufficient time to support community patients, in addition to their responsibilities for hospice inpatients. Overall, staff from all the services involved in providing models A and B referred to the challenge of balancing staff time with high workloads. This often resulted in staff attending to patients’ acute physical symptoms and prioritised them over other palliative care needs.

*5 pm to 9 am, and at the weekends, that’s dependent on the nurses on the palliative care on the hospice unit to answer the phone. If that’s one of our units, [it] has got 10 side rooms around a courtyard. So if there’s three nurses and they’re all with patients, that phone isn’t gonna get answered.* Specialist palliative care medical consultant. Model A. Participant 049: Specialist Palliative Care Consultant.

The lack of timely and responsive care between services was frequently described in models A and B:

*so my last weekend I worked, we called the 111 GPs at 9.30 in the morning. . .By 4.30, they still hadn’t had a visit to the patient. And the patient was in pain.* Model B. Participant: 046 Specialist palliative care nurse.

However, models C and D were able to provide many examples of providing a responsive service:

*Throughout the night. . .the people can access hospice night support and phone them when required.* Model C. Participant 0015: Hospice@home nurse.

Model D described how working with other services and providing integrated care enabled them to provide responsive care which as a standalone service would not be possible.

*it gives us that sort of rapid response service all night. So some of those might be planned visits. In other words, we know we’re going. And others are responsive.* Model D. Participant 0018: Hospice lead.

The multiple steps completed by different services to prescribe, dispense, deliver and administer medication led to delays in relieving distressing symptoms and demanded nursing time to coordinate medication out-of-hours. Many participants (across all four models of care) described the barriers faced in coordination of medicines.

*The out-of-hours community* nurses [after identifying symptom(s) requiring administration of medicines] *would then have to get the drug card to the health centre to be written up* [prescribed by e.g. a GP] *and get the drugs at the same time and then go back to the patient’s house* Model A. Participant 0012: Specialist palliative care medical consultant.

Many saw independent prescribers, such as registered nurse prescribers, as a solution:

*We have very few nurses that can prescribe out in the community. And I think this is a huge gap in our in our requirements because we’re not able to be a one stop service, as we’re relying on somebody else to do that work. And that often doesn’t get done.* Model B. Participant 008: Specialist palliative care nurse.

However, the availability of nurse prescribers was highly variable, with many services (especially in models A and B) not having access to this resource, or only occasionally, depending on which staff were rostered

#### Theme 3: ‘We’re just plugging gaps’: prioritising patient care within limited resources

Healthcare professionals usually strive to fill gaps in care. During the Covid-19 pandemic, services sought and implemented innovations to meet the challenges. A few services also described innovations that enabled quicker access to medicines to manage distressing symptoms, for example ‘grab bags’ containing medicines healthcare professionals could take directly to a patient at home to manage their symptoms in the dying phase, and hospital or hospice dispensing for the community. This enabled patients to receive timely medicines that did not require the coordination of many agencies.

However, participants from models A and B described continuously felt that they were ‘. . . *just plugging gaps, putting sticky plasters on everything all the time*’ (Model A. Participant 049: Specialist palliative care consultant). This piecemeal approach began to have a negative impact on staff. Staff reported working longer hours than contracted; providing services outside their remit; and having to contact staff for help who were not rostered. These gaps were caused by staff shortages (across all models), and in Models A and B, by a lack of skilled professionals available out-of-hours which made crises harder to manage and increased unplanned hospital use. In model A, for example, core services were unavailable during the out-of-hours period. Lack of district or community nursing overnight affected GP workload:

*We don’t have any district nurses from . .eight PM till eight AM. So I think that’s one thing that would enable people to stay at home more than you did because that’s landing on the GPs.* Model A. Participant 0049: Manager.

Participants from models C and D were less likely to describe gaps in care and felt more confident that care was provided when patients and families needed it. Due to the integrated nature of Model D the services were able to work together to ‘plug any gaps’ in care out-of-hours. When one service was unable to work at usual capacity this was quickly resolved with another service usually able to ‘plug the gap’. Model C was generally well resourced out-of-hours by the specialist palliative care teams, and this meant they had less gap to fill.

the*y’ve got really good out-of-hours support. And they’ve got nurse practitioners working who can change prescriptions and things like that overnight.* Model D. Participant:016: Specialist palliative care nurse.

Healthcare professionals also described the personal and emotional challenges they faced in prioritising the out-of-hours needs of patients and carers when they had so many families waiting for care. Healthcare professionals from models A and B spoke about the dissonance between the care they wanted to provide and that which they were able to provide. This caused considerable emotional distress, which was a latent subtheme prevalent in interviews from staff in model A and B.

*they’re covering such a big area with less staff, they can be waiting two hours or more, sometimes, for visits. And I think that’s actually causing a lot of distress* Model A. Participant 0012: Specialist palliative care consultant.

## Discussion

Using the adaption of Bronfenbrenner’s Ecological Systems Theory^23^ enabled us to remain focussed on the patient and family at the centre of provision out-of-hours. The model allowed us to capture the complexity of their changing needs and environment, and the dynamic interactions of the multiple systems involved (see Figure 2). Previous work has examined the out-of-hours care provided to palliative patients and their families at service or team level^14^ or at organisation level, for example the evaluation of Hospice at Home services.^27^ However, this study identified how multiple palliative community healthcare services work together to provide a model of care out-of-hours for patient and families.

The findings indicate how multiple environmental systems impact on the provision of palliative care out of hours, encompassing the microsystem (a person’s needs and characteristics), mesosystem (family/health professionals), exosystem (palliative care service), macrosystem (society and culture) and chronosystem (influences/changes over time).

At the microsystem level, the psychological, physical and spiritual needs of both the patient and their family are considered, which are complex and change over time. Within the mesosystem, we identified the added complexity of healthcare professionals, patients and families needing to navigate many services out-of-hours to provide integrated and coordinated care. Theme one reflected clear fragmentation of care; a longstanding international concern for healthcare services.^28,29^ It is argued that the main barriers to integrated palliative care are policy, education and the size of the specialist workforce.^29^ This paper identifies a model of care (D) which is highly integrated and has the potential to provide high quality out-of-hours care. This integration is enabled by patients and families having a single point of access to a palliative care nurse who could coordinate services on the patient’s and family’s behalf. A scoping review of patient navigation programmes suggests that people can find it challenging to coordinate care and that support from a ‘navigator’ can help address issues of service fragmentation. This is compounded by the presence of additional barriers to accessing services, notably social determinants affecting health such as socio-economic disadvantage.^30,31^ Notably, Model D had further formal structures in place to enhance integrated working such as frequent team meetings and shared electronic patient records between services, and direct telephone numbers for out-of-hours services. This approach is broadly supported by an international systematic review which identified similar facilitators for enhancing integrated working.^32^ In addition, a recent qualitative study of rural palliative care in Norway highlighted the importance of interprofessional working to deliver high quality care this included regular telephone conferences and service locations in close proximity. However, this was impeded by barriers of poor communication between services and a lack of understanding about each other’s roles and competencies^33^. In 2018 a study with healthcare professionals from 5 European countries found that the dominant strategy for fostering integrated palliative care is building core teams of palliative care specialists and extended professional networks rather than developing standardised information exchange and referral pathways.^34^

Challenges at the exosystem level were identified in theme 2; ‘Need for timely skilled management of distressing symptoms’. Pask et al describes how care and patients’ needs can become more complex if needs are not addressed promptly. Supporting this a national survey of 70 Hospice at Home services reported 61% of services reported somewhat or substantial difficulty administering anticipatory medicines by injection in a timely fashion with 97% of providers reporting geography and rurality impacting their ability to provide a responsive service.^27^ Model D facilitated responsive care by having one skilled palliative care nurse leading on coordinating the services. The model D coordinator was aware of the different workloads teams faced, any challenges teams had at the time and the geographical areas healthcare professionals would be covering in each particular shift.

The macrosystem focusses on the wider social context and includes the challenges of funding and prioritisation within limited resources. Prioritising patient care within limited resources therefore sits within the macrosystem. Our findings demonstrate the system barriers when models of care are insufficient due to lack of resource and services are forced to put ‘sticky plasters on everything’. When healthcare professionals from models A and B struggled to meet the needs of patients and families, the findings indicate experiences moral distress, meaning ‘the experience of being seriously compromised as a moral agent in practicing in accordance with accepted professional values and standards’.^35^ Retention of skilled healthcare professionals must become a priority, currently High levels of burnout and staff shortages are commonly reported across healthcare settings with shortages projected to worsen.^36–40^ Sustainable models of out-of-hours care must be invested in to support and retain healthcare professionals.

Previous work has examined the out-of-hours care provided to palliative patients and their families at service or team level^14^ or at organisation level, for example the evaluation of Hospice at Home services.^27^ However, we explored how multiple palliative community healthcare services work together in the UK to provide a model of care out-of-hours for patient and families.

### Strengths and weaknesses/limitations of the study

The main strength of this study is that it encompasses national picture on the provision of community palliative care and exploration between the models of care identified from a range of services. However, there are three main limitations. Firstly, specialist palliative care professionals were overrepresented, contributing to over half of the interviews. Secondly, this study only explores the models of care that patients in receipt of specialist palliative care receive and not the models of care that all people with palliative care needs receive. Thirdly, this study only considers healthcare professionals perspectives. .The impact the patients’ microsystems were not produced from the data analysis. This highlights further the importance of interviewing patients and families to understand their experiences of different models of care and to fully evaluate services.

### Recommendations for practice and policy

The results from this study have enabled the formulation of recommendations for practice and policy on the optimal provision of palliative care out-of-hours. However, these recommendations should be used with caution as further work to understand patients and families’ perspectives and priorities is needed.

1. All community healthcare professionals should have access to direct telephone numbers for other community healthcare providers. Barriers such as needing to contact national health service telephone numbers, to access other providers should be replaced with a local dedicated single point of contact for patients requiring palliative care and the healthcare professionals involved in their care
2. Having formal structures in place to improve integration should be part of the strategic planning of all community healthcare services. Formal strategies should include regular meetings between services, clear agreements about the roles and responsibilities of each team out-of-hours, an identified service in-charge of coordinating all healthcare services involved in palliative patients care, easy access to shared electronic patient records for all services involved in providing a model of out-of-hours care.
3. Out-of-hours community teams should have clear and timely pathways in place for the many steps involved in accessing medicines, including prescribing, dispensing, delivery and administration of medicines.
4. Sustainable models of out-of-hours care (such as model D) must be invested in. Out-of-hours staff vacancies in community healthcare and ensuring adequate staffing of service out-of-hours services needs to become a policy priority to decrease healthcare professionals workloads and prevent moral distress and staff burnout.
5. Healthcare professionals should be encouraged and supported to innovate ways of working that maximises integration between services and addresses common challenges.

## Conclusion

The study identifies four models of community palliative out-of-hours care that display differences in provision and quality of care. The model of care that most strongly facilitates high quality care is highly integrated and comprises of: a single point of access, staffed by experienced palliative care healthcare professionals, with access to shared electronic patient records and is positioned and skilled to coordinate care across all involved services. Out-of-hours care is *‘never only one service’*, detailed coordination and formal structures and processes are required to ensure effective and efficient integration and achieve high-quality care.

## Supplemental Material

sj-docx-1-pmj-10.1177_02692163241302671 – Supplemental material for Community out-of-hours palliative care – ‘It’s a patchwork of services’: A qualitative study exploring care provision Supplemental material, sj-docx-1-pmj-10.1177_02692163241302671 for Community out-of-hours palliative care – ‘It’s a patchwork of services’: A qualitative study exploring care provision by Alice M Firth, Joanna Goodrich, Inez Gaczkowska, Richard Harding, Fliss EM Murtagh and Catherine J Evans in Palliative Medicine
