# Supplementary material for: Community out-of-hours palliative care – ‘It’s a patchwork of services’: A qualitative study exploring care provision
Source: Palliat Med. 2024 Dec 11;39(2):245–55. doi: 10.1177/02692163241302671 (PMC11776347; doi:10.1177/02692163241302671)
Supplement: sj-docx-1-pmj-10.1177_02692163241302671 – Supplemental material for Community out-of-hours palliative care – ‘It’s a patchwork of services’: A qualitative study exploring care provision [file sj-docx-1-pmj-10.1177_02692163241302671.docx]

**Supplementary material 1: Interview Topic Guide**

**Introductory questions:**

**Please could you tell us what your role is and how long you have worked in palliative care**

**Can you please describe how out-of-hours care is defined by your service?**

Probe: What times do you classify as out-of-hours?

**Part one:**

**Please think of a recent, fairly typical patient receiving services at home, and talk me through which services they would access out-of-hours for help and support?**

*Probe: what are the patients/families contacting these different services for?*

*Probe: How do the patient/family contact these services?*

*Probe: Who or which service would be the patient/family member’s first point of contact?*

*Prompt: GP, community nurses, Marie Curie/ Macmillan nurses or any other services you work with routinely?*

*(Probe: Which professionals respond?)*

**How is this support similar or different if it is the family carer who is in crisis/ finding it hard to cope***? (practical/ emotional)*

**Out of hours, are the services for patients and families the same or different in the last few days of life If different, how?**

**How is the prescription for, access to, and administration of medicines managed out-of-hours? Is this the same for all patients?**

*Probe: Is this the same for all patients under your care*

**Part two:**

**Can you tell me about how you work with other services to deliver care out-of-hours?**

**Can you please describe how the out-of-hours services are funded/commissioned?**

*Probe: Do you work with social care providers and how?*

**How do services communicate, both out-of-hours, and afterwards during usual hours?**

*Probe: If the patient’s needs change, such as worsening symptoms declining function out-of-hours? Which other services would you communicate this to and how would it be communicated?*

*Probe: What is the level of knowledge among healthcare providers about the different OOH services and roles? How is this knowledge shared?*

**Do you think patients and families experience care as integrated, or ‘seamless’? What does not work so well?**

**What would you say the biggest challenge is out-of-hours?**

**Part three:**

**As part of this study we have conducted a systematic review of evidence and from this we have developed a categorisation of the different services that patients and families under specialist palliative care receive. We would like to understand which of these services patients under your specialist palliative care service receive. A patient and family may receive one or more than one of these services.**

*(Typologies to be presented on A4 sheet-see Figure 1.– sent via e-mail alongside confirmation of interview time but also share screen if necessary)*

**Can you indicate which one applies for your specialist palliative care service?**

**Can you also indicate which other services a patient will receive in your area?**

*Probe:* Do you think the way we have categorised these services works well or not? If not, why not?

*Probe: Would you categorise the services differently?*

*Probe: Do you think we are missing anything? Would you add an additional dimension (currently we have 24/7, SPC or generalist and hands on or advisory)? If so what would this be?*

**Closing question: Would you like to tell us anything further about the out-of-hours care you provide or the categories of services we have developed?**


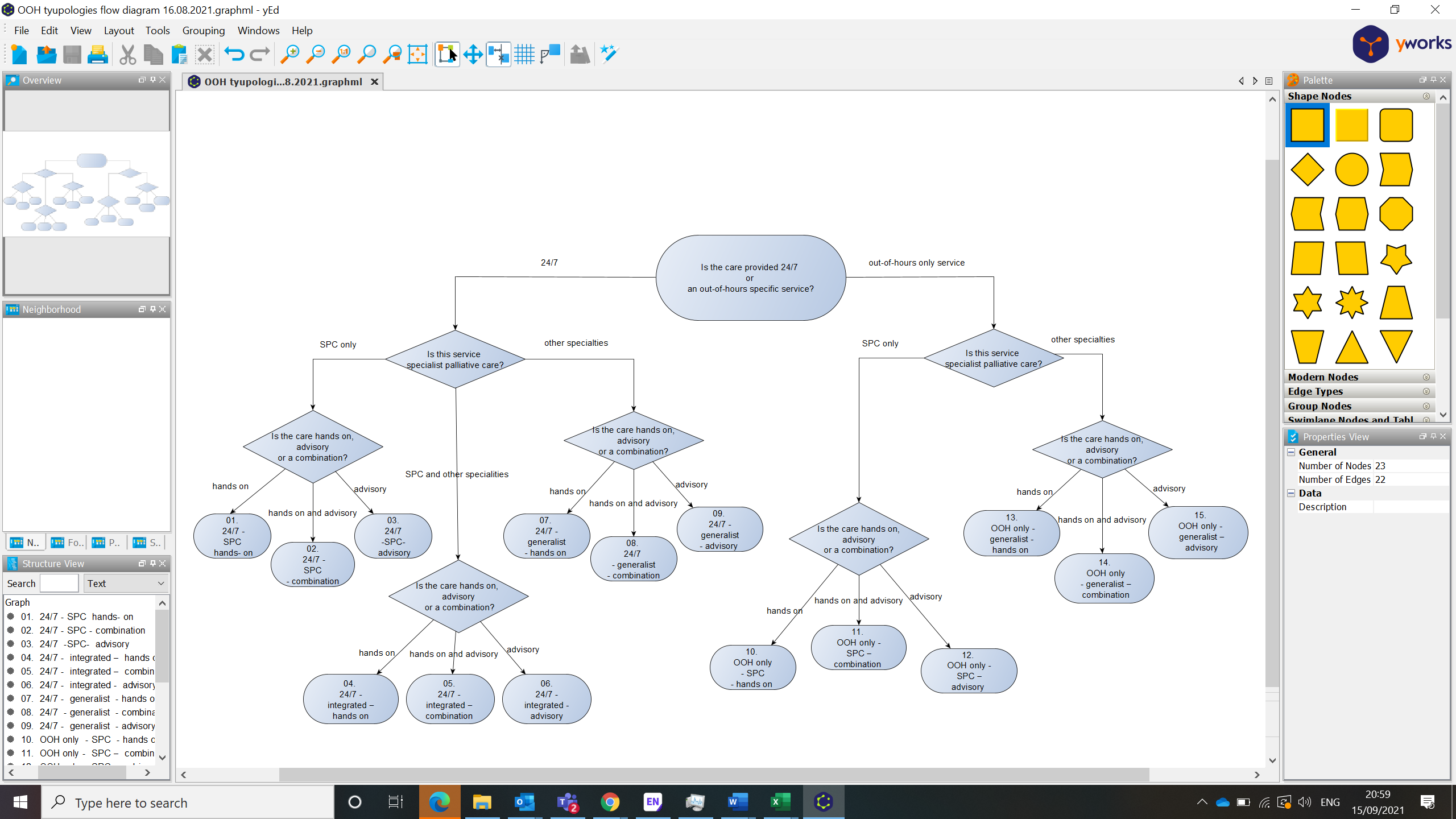
Figure 1: Multi-dimensional typologies of out-of-hours service delivery

| **Supplementary material 2:** | **Themes** | | |
| --- | --- | --- | --- |
|  | **‘It’s never one service’: challenges of coordination of care across multiple services** | **Need for timely skilled management of distressing symptoms** | **‘We’re just plugging gaps ‘: prioritising patient care within limited resources** |
| **Theme definitions and characteristics:** | Coordination of care includes the coordination of internal team as well as coordinating with several external agencies(e.g. district nursing, community nursing teams, specialist palliative care teams, charitable teams, GPs and pharmacies). Coordination of care involves the planned structures and processes to enable teams to work together.  This theme includes the challenges of coordinating several services out-of-hours.  When services are well coordinated, patients and families receive continuity and better quality of care.  A serious challenge to providing high quality care is the limited staffing of out -of-hours services. | The importance of ensuring patients and families receive responsive care out-of-hours to relieve distressing symptoms when the care and treatment is not keeping up.  This includes healthcare professionals’ prioritising provision of clear and simple access to services out-of-hours for patients and families that are responsive to changing needs. For example, models of care that have a single point of contact for out-of-hours palliative care services with skilled practitioners able to assess care needs, coordinate services and provide timely and responsive care.  Patient and family’s need to receive medicines to relieve distressing unexpected symptoms in a timely manner. The numerous stages of accessing medicines out-of-hours can cause delays e.g. assessment, prescribing, delivery of medicines, and their administration.  It is distressing for patients, families, and staff when care is delayed. | Healthcare professionals involved in out-of-hours care usually strive to meet needs; ‘filling the gaps’ in care provision by going beyond their remit or seeking innovations. For example, contacting staff who are not rostered for advice, staff working longer hours than contracted, providing services beyond commissioned brief.  However, it was not always possible for staff to ‘plug the gaps’. This could result in staff distress when staff were unable to deliver the level of care required because of resource constraints.  Plugging the gaps is a response to insufficient healthcare resource to meet the needs of patients and families. |
| **Sub themes** | Continuity of care  Anticipating/ Planning care for out-of-hours period  Formal / integrated working structures | Responsiveness of a service  Face to face care  Timely access to Medicines  Case load versus resource  Skilled professionals | Beyond the remit of healthcare professionals’ roles  Geographic variation  Innovations  Impossible tensions  Staff distress |
